# A spatially filtered multilevel model to account for spatial dependency: application to self-rated health status in South Korea

**DOI:** 10.1186/1476-072X-13-6

**Published:** 2014-02-27

**Authors:** Yoo Min Park, Youngho Kim

**Affiliations:** 1 Department of Geography, Korea University, Anam-dong, Seongbuk-gu, Seoul, Korea; 2 Department of Geography Education, Korea University, Anam-dong, Seongbuk-gu, Seoul, Korea

**Keywords:** Self-rated health status, Multilevel model, Eigenvector spatial filtering, Spatial dependency

## Abstract

**Background:**

This study aims to suggest an approach that integrates multilevel models and eigenvector spatial filtering methods and apply it to a case study of self-rated health status in South Korea. In many previous health-related studies, multilevel models and single-level spatial regression are used separately. However, the two methods should be used in conjunction because the objectives of both approaches are important in health-related analyses. The multilevel model enables the simultaneous analysis of both individual and neighborhood factors influencing health outcomes. However, the results of conventional multilevel models are potentially misleading when spatial dependency across neighborhoods exists. Spatial dependency in health-related data indicates that health outcomes in nearby neighborhoods are more similar to each other than those in distant neighborhoods. Spatial regression models can address this problem by modeling spatial dependency. This study explores the possibility of integrating a multilevel model and eigenvector spatial filtering, an advanced spatial regression for addressing spatial dependency in datasets.

**Methods:**

In this spatially filtered multilevel model, eigenvectors function as additional explanatory variables accounting for unexplained spatial dependency within the neighborhood-level error. The specification addresses the inability of conventional multilevel models to account for spatial dependency, and thereby, generates more robust outputs.

**Results:**

The findings show that sex, employment status, monthly household income, and perceived levels of stress are significantly associated with self-rated health status. Residents living in neighborhoods with low deprivation and a high doctor-to-resident ratio tend to report higher health status. The spatially filtered multilevel model provides unbiased estimations and improves the explanatory power of the model compared to conventional multilevel models although there are no changes in the signs of parameters and the significance levels between the two models in this case study.

**Conclusions:**

The integrated approach proposed in this paper is a useful tool for understanding the geographical distribution of self-rated health status within a multilevel framework. In future research, it would be useful to apply the spatially filtered multilevel model to other datasets in order to clarify the differences between the two models. It is anticipated that this integrated method will also out-perform conventional models when it is used in other contexts.

## Background

To analyze both effects of individual and neighborhood factors on individual health outcomes, many previous health-related studies utilized multilevel models that can analyze two- (or more) level independent variables in tandem [1-6]. These studies analyzed various health outcomes, such as infant mortality [1], a low birth weight [2], preterm birth [3], late-stage breast cancer [4], children’s health-related quality of life [5], and tuberculosis incidence [6], using aggregated data in common, such as county-level, census tract-level, and postal code-level data to represent neighborhood-level variables. The studies, however, do not take into account underlying spatial dependency across neighborhoods; thus their multilevel analyses results are potentially misleading in cases where data exhibit spatial dependency. Spatial dependency in health-related data indicates that health outcomes in nearby neighborhoods are more similar to each other than to those in distant neighborhoods. In other words, these studies only consider within-neighborhood correlation (i.e., correlation between individuals within the same neighborhood) using a hierarchical setting, but fail to account for potential between-neighborhood correlation.

According to Jerrett et al. [7], spatial dependency of health outcomes among nearby neighborhoods may arise from similar socioeconomic (e.g., health facilities and services) and natural environmental conditions (e.g., air quality). For example, catchment areas for health facilities may encompass a broader area, thereby transcending localized administrative boundaries. In terms of local environment, disease risks from air pollution tend to be similar among closer neighborhoods because their local wind direction and/or road conditions (and environmental and traffic policies) are more likely to be similar; as a result, residents of those neighborhoods are exposed to similar types and concentrations of atmospheric pollutants [7-9]. However, the non-spatial multilevel model cannot address this spatial dependency because the method typically assumes that neighborhoods (i.e., spatial units) are statistically independent of each other [10]; thus multilevel models have been criticized as non-spatial and unrealistic [10-13].

Based on the notion of spatial dependency of health outcomes, some researchers used both a non-spatial single-level linear model ignoring spatial dependency (i.e., linear models estimated with ordinary least squares or weighted least squares) and a spatial autoregressive model (SAR) considering spatial dependency, and compared the two methods [9,14]. The authors found that non-spatial single-level models and the SAR models provided different regression results depending on the presence of spatial dependency. These two studies, however, made limited attempts to model individual characteristics when using spatial models, because they used only aggregated variables. Studies that analyze health outcomes solely via aggregated data using a single-level spatial model cannot fully explain factors that truly influence individual health outcomes [15].

A few researchers have tried to incorporate a geographical perspective into the multilevel setting in various ways to take into account both the multilevel framework and spatial effects. Some studies attempting to address spatial dependency in residuals of multilevel models employed spatial lag regression model specifications [16,17]. In the spatial lag regression model, the spatial autoregressive parameter is denoted as *ρ*, which indicates the intensity of spatial dependency. Another study [18] used multilevel models with geographically weighted regression (GWR) developed by Fotheringham et al. [13] to consider a spatially varying relationship between neighborhood factors and obesity. GWR allows researchers to estimate varying regression parameters over space. However, in some cases, there can still be spatial dependency after GWR is used, although this method may mitigate spatial dependency by considering spatial variation to some degree; this can influence the regression results considerably. In addition, according to Wheeler and Tiefelsdorf [19], GWR’s R^2^ goodness of fit tends to be high when residuals have high spatial dependency. Therefore, GWR should be used as an exploratory tool for understanding spatial variation rather than a statistically stable method for addressing spatial dependency.

As discussed above, limited attention has been paid within the literature to integrating multilevel models and spatial regression models. However, these two approaches should be used in combination because the objectives of both methods are important in health-related analyses. Thus, it is increasingly necessary to integrate multilevel models and spatial regression models, especially the eigenvector spatial filtering method, an advanced approach to addressing spatial dependency in datasets. Compared to spatial lag regression (or SAR) model specifications, which present only one parameter of global spatial component, the greatest advantage of eigenvector spatial filtering used in this paper is to visualize a spatial structure in a map form by decomposing it into smaller-scale spatial patterns or local clusters with a set of eigenvectors [20,21]. This trait could provide a better understanding of how health phenomena are distributed across the space. Additionally, because the spatial filtering technique can be applied to a generalized linear model specification based on the binomial or Poisson probability models, it is more flexible than the spatial lag regression (or SAR) model, which requires normalizing factor computation [22]. Compared with GWR, which has an inherent problem of multicollinearity among local regression coefficients [19], the spatial filtering method is more statistically reliable because eigenvectors generated in filtering procedure are mutually orthogonal, which indicates the absence of multicollinearity issues.

Griffith’s study [22] showed the possibility of combining hierarchical generalized linear models with spatial filtering method as a disease mapping technique. Based on this idea, the present study presents how multilevel modeling components can be linked to the spatial filtering framework by showing an integrated formulation and uses self-rated health status in South Korea to investigate whether an integrated “spatially filtered multilevel model” generates a more robust regression results than a conventional multilevel model.

This study first identifies whether spatial dependency exists within neighborhood-level residuals in the multilevel model. Where spatial dependency is detected, the eigenvector spatial filtering technique is applied to the multilevel model to control for spatial dependency. The study then compares the explanatory power of the models and the regression results between the conventional model and the spatially filtered model.

## Methods

### Data and variables

Data are obtained from the following sources: (1) the 2009 Community Health Survey (CHS) of South Korea; (2) the e-Regional Indicators (2009) provided by Statistics Korea; and (3) the Korean Deprivation Index (KDI) designed by Yoon [23]. The CHS is a survey of health outcomes among adults aged 19 or older, conducted by the Korea Centers for Disease Control and Prevention. A dependent variable, EQ-5D index (EuroQol-5 Dimension [24]), is obtained from the CHS. The EQ-5D index indicates one of the measures of self-rated health status. This index comprises five dimensions (mobility, self-care, usual activities, pain/discomfort, and anxiety/depression) that are measured by means of a three-point scale (no problems; some problems; extreme problems). Respondents are asked to assess their own health status by selecting the most appropriate indicator for each dimension. Thus, based on these responses, a total of 3^5^ types of self-rated health status are produced. Each type has different EQ-5D values that enable researchers to compare health status between regions or countries [25,26]. A higher value indicates that a respondent perceives him/herself healthier. Based on CHS’ EQ-5D questionnaire responses, the study employs a weighted model^a^ developed by Kang et al. [27] to calculate a Korean EQ-5D index. Table 1 provides descriptive statistics for the Korean EQ-5D index. In order to minimize the impact of variability in age distribution across the country, the study included individuals aged 60 and older. From 61,817 respondents, the average of the Korean EQ-5D index is 0.783 and standard deviation is 0.261 (range -0.229 to 1.0).

**Table 1:** Descriptive statistics for a dependent variable and independent variables

	**N**	**%**	
***Individual-level variables*** (n = 61817)			
Sex			
Males	26116	42.2	
Females	35701	57.8	
Employment status			
Employed	24508	39.7	
Unemployed	37293	60.3	
Perceived levels of stress			
High level	13140	21.3	
Low level	48649	78.7	
	**Mean**	**Standard dev.**	**Range**
Monthly household income (US$)	1382.1	1988.4	0.0 – 99553.6
***Neighborhood-level variables*** (n = 223)			
Korean Deprivation Index (KDI)	0.3	0.9	-1.5 – 1.7
The number of doctors per 1000 people	2.2	2.0	0.6 – 20.7
Degree of the Local Governments’ Financial Independence (LGFI)	65.1	9.5	33.7 – 91.4
***Dependent variable***			
EQ-5D index	0.783	0.261	- 0.229 – 1.000

**Figure 1:**
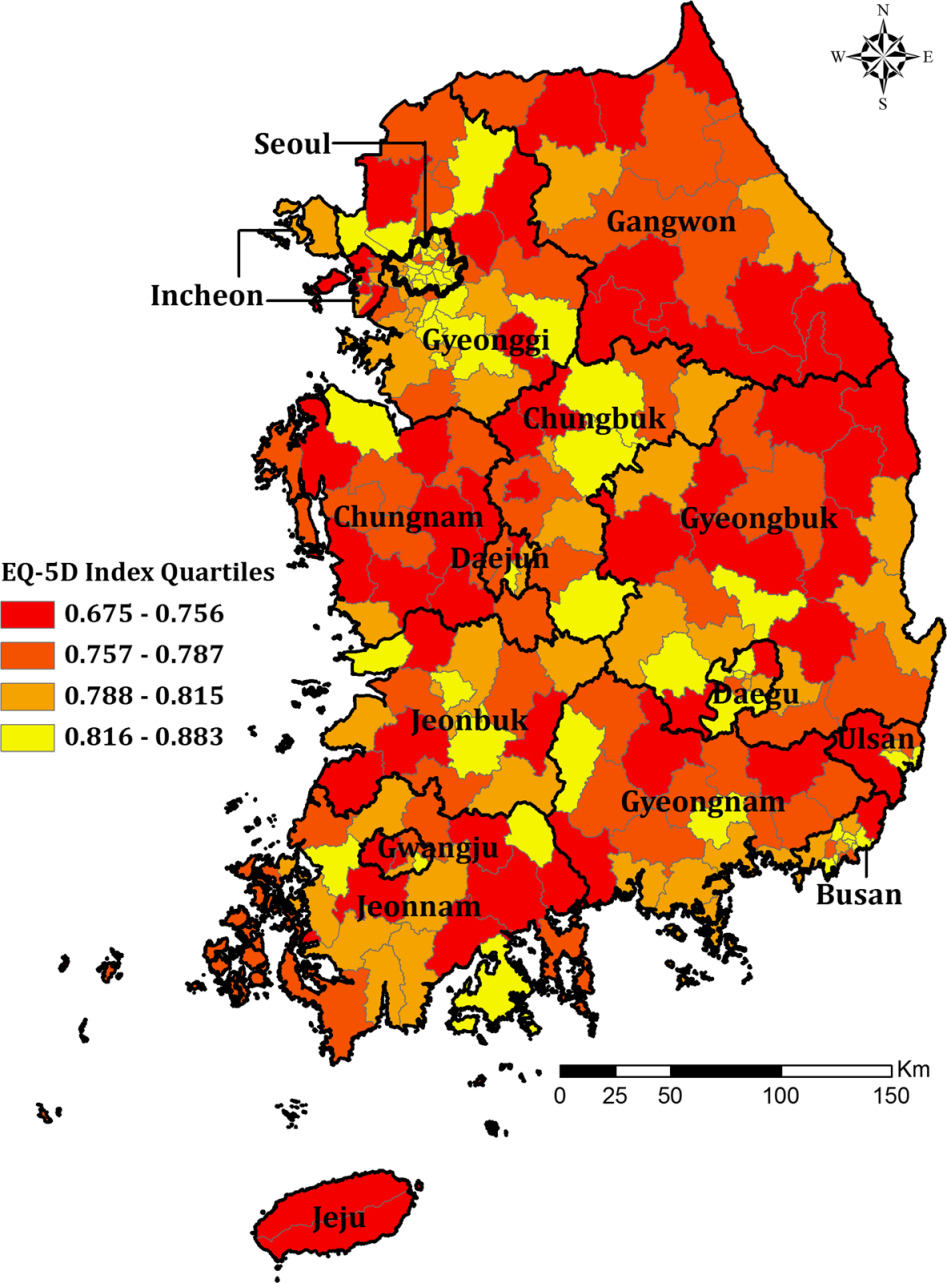
**Self-rated health status by census tracts, South Korea** ***.***

To explore how self-rated health status varies across the study area, census tracts are classified into four quartiles depending on neighborhood-level EQ-5D values: “Very low” (first quartile: 0.675 – 0.756), “Low” (second quartile: 0.757 – 0.787), “Average” (third quartile: 0.788 – 0.815), and “High or very high” (fourth quartile: 0.816 – 0.883). The values are visualized as a choropleth map (Figure 1).

Figure 1 shows how self-rated health status is more similar to that in nearby neighborhood census tracts than that in distant neighborhoods. This is because nearby neighborhoods are likely to have similar demographic and socioeconomic characteristics (e.g., sex, age, race, income, language, and religion) and political resources within a larger citywide system [28,29]. In South Korea, development policies have focused more on rapid economic growth than the distribution of accumulated wealth, resulting in serious regional disparities in health status across the country. For example, most districts in Seoul, Korea’s largest metropolitan area, show high self-rated health status (Figure 1). This is because the Seoul metropolitan area has sufficiently dense infrastructure provision for a healthy environment to ensure good accessibility to health services [30]. In contrast, many provincial cities in non-metropolitan areas excluded from the benefits of economic development, such as Gangwon, Chungnam, and Gyeongbuk show low self-rated health status.

The CHS also provides individual-level variables such as sex, employment status, perceived levels of stress, and monthly household income. Among these, sex (0 = males; 1 = females), employment status (0 = employed; 1 = unemployed), and perceived levels of stress (0 = people with high perceived levels of stress; 1 = people with low perceived levels of stress) are binary. Monthly household income is a continuous variable. Descriptive statistics for the independent variables are summarized in Table 1.

The neighborhood-level variables consist of the KDI [23], the doctor-to-resident ratio (number of doctors per 1,000 population), and the degree of the local governments’ financial independence (LGFI). The KDI is based on eight census indicators reflecting neighborhood socioeconomic levels, such as the proportions of households that are: without car ownership; in a low social class; comprised of elderly people, etc. The number of doctors per 1,000 population and LGFI were obtained from e-Regional Indicators (2009). LGFI refers to the local government’s level of autonomy to raise and use financial funds. This ability facilitates implementation of welfare policy, such as providing healthy residential environment or enhancing health care services. The ratio of physicians to residents reflects accessibility to health care services. Descriptive statistics for neighborhood-level variables are provided in Table 1.

### Multilevel model

When analyzing both individual and neighborhood variables in tandem, a multilevel model is generally more appropriate than an ordinary single-level regression model because it enables researchers to deal with hierarchical structure of variables [31]. The multilevel model assumes that individuals (i.e., lower hierarchy) belonging to a particular neighborhood (i.e., higher hierarchy) are not independent of each other because they are presumed to share the similar characteristics of that neighborhood; thus the model considers intra-neighborhood correlation.

Model construction begins with analyzing a ‘null’ model, which is the simplest model and uses no independent variable. The null model includes distinct types of variance of the dependent variable, such as within-neighborhood and between-neighborhood variances [32]. Based on this null model, an Intra-class Correlation Coefficient (ICC) is calculated, which guides how the null model should be extended further. The ICC is the ratio between the between-neighborhood variance and the sum of both within-neighborhood and between-neighborhood variances. A high ICC indicates that between-neighborhood variance is not negligible, and thus a multilevel model should be employed to explain the inter-neighborhood dynamics.

The null model is then extended to a more advanced multilevel model by adding independent variables at the individual- and neighborhood-levels. The two-level Equation 1 is expressed as follows [32]:

(1) $$\begin{array}{c} \mathrm{Individual}-\mathrm{level}:Y_{\mathrm{ij}}=\beta_{0\;j}+\beta_{1j}X_{\mathrm{ij}}+r_{\mathrm{ij}}\\ \mathrm{Neighborhood}-\mathrm{level}:\beta_{0\;j}=\gamma_{00}+\gamma_{01}Z_{j}+u_{0\;j},\beta_{1j}=\gamma_{10}+u_{1j} \end{array}$$

Here, *Y*_*ij*_ represents the value of the dependent variable of the *i* th individual in neighborhood *j*, while X_*ij*_ and Z_*j*_ indicate the independent variables at different levels. In other words, X_*ij*_ includes data about the individuals in neighborhood *j*; Z_*j*_ contains data about the neighborhoods. *β*_0 *j*_ and *β*_1*j*_ are the individual-level intercept and slope, respectively, in neighborhood *j. r*_*ij*_ indicates the error term at the individual-level (i.e., within-neighborhood variance). *γ*_00_ denotes the average of the dependent variable *Y*_*ij*_, controlling for the neighborhood-level variables Z_*j*_; *γ*_01_ is the slope of the neighborhood-level variables Z_*j*_; and *γ*_10_ indicates the overall value of slope at the individual-level, controlling for the neighborhood-level variables Z_*j*_. Lastly, *u*_0 *j*_ and *u*_1*j*_ are error terms at the neighborhood-level (i.e., between-neighborhood variance). In the framework of multilevel modeling, an intercept is assumed to be inconsistent if the neighborhood averages of a dependent variable differ between neighborhoods. Similarly, when effects of independent variables on the dependent variable vary across neighborhoods, the slopes of each neighborhood are expected to deviate from their average.

### Eigenvector spatial filtering

Proposed by Griffith [33], an eigenvector spatial filtering technique handles spatial dependency within ordinary single-level regression by utilizing a linear combination of eigenvectors. Eigenvectors function as synthetic explanatory variables expressing underlying spatial structures of the regression model [20]. This method allows one to visualize local spatial clusters in a map form. Because eigenvectors are always independent of each other, the associated spatial structures are thus regarded as being distinct.

From the perspective of eigenvector spatial filtering, an ordinary single-level regression applied to spatial datasets consists of two parts: (1) a systematic trend explained by independent variables, and (2) unexplained random errors that are often spatially autocorrelated [34,35]. That is to say, the eigenvector spatial filtering technique can capture a spatial signal from unexplained random errors, which in turn reinforces the independence of the error term [35,36]. This is expressed numerically in Equation 2:

(2) $$Y=X\beta +\epsilon^{*}=X\beta +\underset{\epsilon^{*}}{\underset{︸}{E\gamma +\xi }}$$

where **X***β* refers to the systematic trend, while ϵ^*^ is the *n*-by-1 spatially autocorrelated error vector. **X** denotes the *n*-by-*k* data matrix (i.e., *n* number of observations and *k* number of independent variables); *β* indicates the *k*-by-1 parameter vector corresponding to the independent variables. **E***γ* is the spatial signal captured by selected eigenvectors **E**. The dimension of **E** is *n*-by-*p* (i.e., *n* number of observations and *p* number of selected eigenvectors), and *γ* is the *p*-by-1 parameter vector corresponding to the selected eigenvectors. Lastly, *ξ* is the *n*-by-1 spatially-independent error vector.

When generating eigenvectors, two different spatial processes are considered: (1) simultaneous autoregressive (SAR); and (2) spatial lag [35]. These processes may generate different analytical results due to their differing model structures; for further details, see the study by Tiefelsdorf and Griffith [35]. The present study deals only with eigenvectors for the SAR process.

Eigenvectors for the SAR process, {e_1_, e_2_, ⋯, e_n_}_SAR_, are extracted from a transformed spatial weight matrix as follows:

(3) $$\;{\left\{e_{1},e_{2},\cdots ,e_{n}\right\}}_{\mathrm{SAR}}\;\equiv \;\mathrm{evec}\left[M_{\left(X\right)}\frac{1}{2}\left(V+V^{T}\right)M_{\left(X\right)}\right]$$

where a projection matrix **M**(x) ≡ **I - X(X**^T^**X)**^-1^**X**^T^; **I** represents an *n***-**by-*n* identity matrix; **X** is an *n***-**by-*k* matrix including *n* number of objectives and *k* number of independent variables. A subset of {e_1_, e_2_, ⋯, e_n_}_SAR_ is denoted by **E**_SAR_, which contains only selected eigenvectors. This set of eigenvectors can be introduced in a model as spatial proxies to ‘filter out’ spatial dependency [35].

Eigenvectors are selected in a stepwise manner, and the selection procedure is repeated until the value of Moran’s *I*^b^ (an indicator of a strength of spatial dependency) approaches a pre-determined threshold (e.g., |z(Moran’s *I*)| < 0.1). Each eigenvector, owing to their mutual orthogonality, shows its unique spatial patterns and different degrees of spatial dependency. The first selected eigenvector has the highest Moran’s *I* value and therefore accounts for the largest proportion of the overall spatial dependency. The second eigenvector has the second-highest Moran’s *I* value, and is uncorrelated with the first one [20]; similarly, the *n*th eigenvector is considered to have the *n*th-highest Moran’s *I* value, expressing the *n*th-largest proportion of the spatial dependency.

### Spatially filtered multilevel model

Equation 1 of the conventional multilevel model can be rearranged as follows:

(4) $$Y_{\mathrm{ij}}=\underset{\mathrm{fixed}\;\mathrm{effects}}{\underbrace{\gamma_{00}+\gamma_{01}Z_{j}+\gamma_{10}X_{\mathrm{ij}}}}+\underset{\mathrm{random}\;\mathrm{effects}}{\underbrace{u_{0\;j}+u_{1j}X_{\mathrm{ij}}+r_{\mathrm{ij}}}}$$

Basically, this multilevel model can be divided into two parts, representing fixed effects (that are modeled in a multileveled manner), and random effects (that are unexplained and often spatially autocorrelated). If this model is corrected by the eigenvector spatial filtering technique, the spatial signal can be introduced as follows:

(5) $$Y_{\mathrm{ij}}=\overset{\text{fixed effects}}{\underset{X\beta (\text{systemic trend})}{\overset{⏞}{\underset{⏟}{\left(\gamma_{00}+\gamma_{01}Z_{j}+\gamma_{10}X_{\mathrm{ij}}\right)}}}}+\overset{\text{spatially autocorrelated random effects}}{\overset{⏞}{\underset{E\gamma (\text{spatial signal})}{\underset{⏟}{\left(\gamma_{0}+\gamma_{1}e_{1j}+\cdots +\gamma_{n}e_{\mathrm{nj}}\right)}}+\underset{\xi (\text{white-noise})}{\underset{⏟}{\left({\mathrm{u'}}_{0j}+{\mathrm{u'}}_{1j}X_{\mathrm{ij}}+r\mathrm{ij}\right)}}}}$$

This integrated model, entitled ‘spatially filtered multilevel model,’ regards the fixed effects in the multilevel model as identical to the systematic trend **X***β* in the framework of eigenvector spatial filtering. In this model, a linear combination of eigenvectors **E**γ is included as a spatial proxy to separate the spatial signal from the spatially autocorrelated random effects at the neighborhood-level (*u*_0*j*_ + *u*_1*j*_*X*_*ij*_), leaving only a white-noise $u_{0j}^{'}+u_{1j}^{'}X_{\mathrm{ij}}$ within them. This filtering process results in unbiased regression results that improve the explanatory power of the model.

All analyses are conducted in the *R* environment. The ‘lme4’ package [37] is used for the multilevel model run, and the ‘spdep’ package [38] is employed for the ‘SpatialFiltering’ function for the eigenvector spatial filtering.

## Results

### Results of the conventional multilevel model

The null model finds that the variance at neighborhood-level is 2.3% (ICC = 0.023). This indicates that 2.3% of the total variance in self-rated health status arises from inter-neighborhood dynamics. Given that a health outcome itself is generally influenced more by individual factors than by neighborhood characteristics, it is reasonable that variance at individual-level is much larger than that at neighborhood-level. The 2.3% variance at neighborhood-level should be regarded with some caution, because Kreft and de Leeuw pointed out that for a sufficiently large number of samples, even a small ICC (for example, 1%) could considerably affect the degree of significance [31].

To identify the effects of independent variables on individual health status, the individual-level model (hereafter, Level-1 model) is then designed by adding individual-level variables to the null model. An intercept for each independent variable in this study is assumed to be random across the study area. Except for the slope for the monthly household income variable, a slope for each independent variable is regarded as fixed for simplicity of modeling. As shown in Table 2, the Level-1 model yields much lower Akaike Information Criterion (AIC) compared to the null model, indicating a better model fit [39]. All individual-level variables (sex, employment status, perceived levels of stress, and monthly household income) are significantly associated with individual self-rated health status. These variables are found to account for 22% of variance at individual-level and 31% of variance at neighborhood-level. The reason why the Level-1 model partially explains variance at neighborhood level—despite it not including neighborhood-level variables—is that regression analyses are performed separately for each neighborhood.

**Table 2:** Estimation results for the conventional multilevel model and the spatially filtered multilevel model

**Variables**	**Null model**	**Level-1 multilevel model**	**Level-2 multilevel model**	**Spatially filtered multilevel model**
***Individual-level variables***				
Sex (male:0; female:1)	-	– 49.88***	– 49.65***	– 49.69***
Monthly household income	–	0.10***	0.10***	0.09***
Employment status (employed:0; unemployed:1)	–	–134.10***	–134.90***	–135.30***
Perceived levels of stress (high:0; low:1)	–	154.60***	155.60***	155.70***
***Neighborhood–level variables***				
Korean Deprivation Index (KDI)	–	–	–23.82*	–15.51*
The number of doctors per 1000 people	–	–	4.85*	2.60*
Degree of the Local Governments’ Financial Independence (LGFI)	–	–	0.98	0.16
***Random effects***				
Variance at individual–level	66725	52226	52225	56013
Between monthly household income variance	–	0.0039	0.0036	0.0011
Variance at neighborhood–level	1591	1102	1062	555
Constant	785.31***	761.40***	747.00***	770.70***
Eigenvector selection	–	–	–	8 eigenvectors
Moran’s *I* of neighborhood–level residuals	–	–	0.101*	0.005
AIC	861942	830665	830650	830549
Log–likelihood	– 447328	– 415324	– 415314	– 415254

***p < =0.001 *p < =0.05.

For the next step, both individual-level and neighborhood-level variables are added together in the neighborhood-level model (hereafter, Level-2 model). By introducing neighborhood-level variables, a further 2% of variance at neighborhood-level is explained compared with the Level-1 model. This suggests that neighborhood factors explicitly influence the individuals’ self-rated health status. The Level-2 model shows the lowest AIC and the highest explanatory power among the three models. Like the Level-1 model, all individual-level variables remain significant (p < 0.001). Of the three neighborhood-level variables, only two variables, KDI and the doctor-to-resident ratio, are statistically significant (p < 0.05) (Table 2).

### Results of applying eigenvector spatial filtering

Before applying the eigenvector spatial filtering method, we tested for spatial dependency between neighborhood-level residuals in the multilevel model and found this to be significant (Moran’s *I* = 0.101; p < 0.05). Hence, it is necessary to eliminate this spatial dependency by applying the eigenvector spatial filtering.

Eigenvectors in this study are extracted from a transformed spatial weight matrix based on topological adjacency, so-called a “Queen” criterion*—*if two areas share a boundary or a vertex, the entity of the spatial weight matrix is coded as 1, and otherwise, 0. As an eigenvector selection algorithm, this study uses a Moran’s *I* minimization scheme [35].

**Figure 2:**
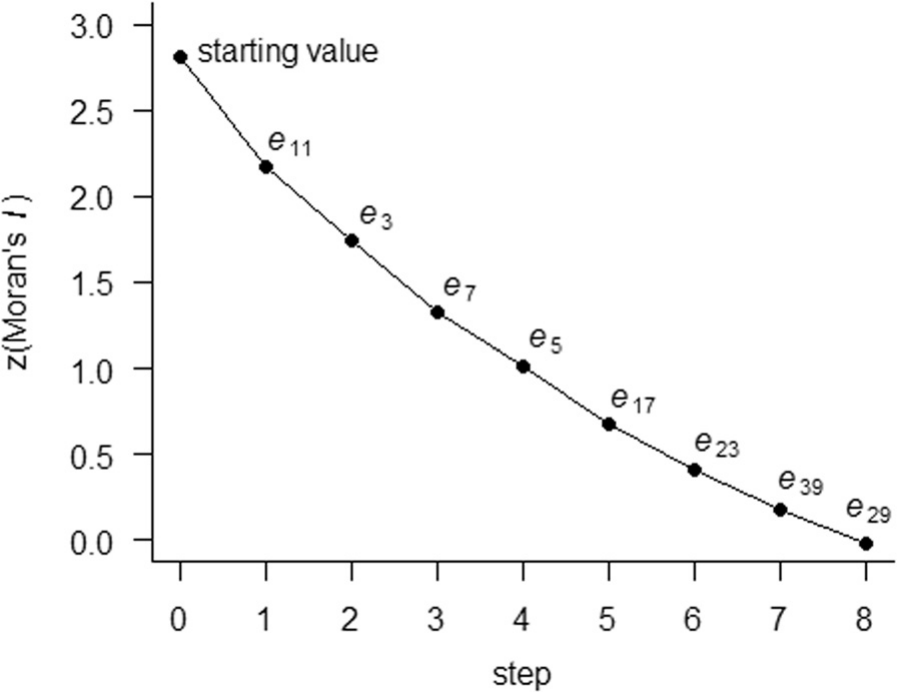
**Reduction of Moran’s *I *** **by eigenvector spatial filtering procedure.**

Figure 2 shows that by adding eigenvectors to the model, the degree of spatial dependency becomes reduced to the threshold (|z(Moran’s *I*)| < 0.1). This is because selected eigenvectors explain spatial dependency as synthetic variables. A group of 8 eigenvectors (*e*_11_, *e*_3_, *e*_7_, *e*_5_, *e*_17_, *e*_23_, *e*_39_ and *e*_29_) are finally selected. The first selected eigenvector *e*_11_ explains the greatest proportion of spatial dependency (Figure 2).

**Figure 3:**
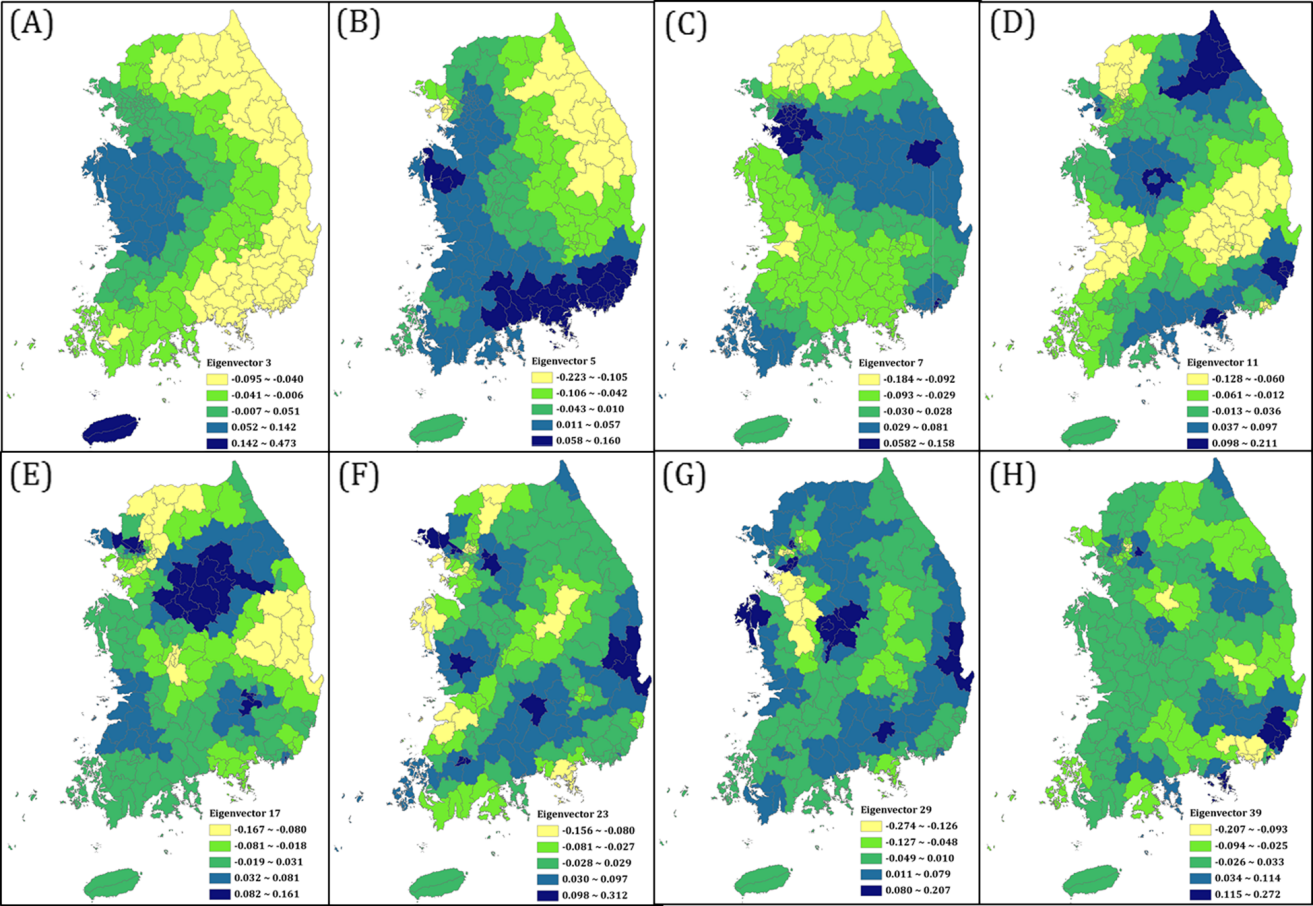
**Spatial patterns of selected SAR eigenvectors.***Notes*: **(A)** First selected eigenvector *e*_11_. **(B)** Second selected eigenvector *e*_3_. **(C)** Third selected eigenvector *e*_7_. **(D)** Fourth selected eigenvector *e*_5_. **(E)** Fifth selected eigenvector *e*_17_. **(F)** Sixth selected eigenvector *e*_23_. **(G)** Seventh selected eigenvector *e*_39_. **(H)** Eighth selected eigenvector *e*_29_.

Selected eigenvectors are illustrated in Figure 3, all of which portray positive spatial dependency patterns. The first four eigenvectors exhibit explicit local clusters related to positive spatial dependency across the study area. Given that the first sequenced eigenvector represents more noticeable cluster than those later in the series, *e*_11_ displays the most prominent local cluster pattern, as shown in Figure 3-A.

## Discussion

The spatially filtered multilevel model presents unbiased regression results and yields a lower AIC than the conventional multilevel model. Both analyses present similar regression parameters and the same parameter signs (Table 2). In this study, addressing spatial dependency has little effect on the fixed effects, whereas it improves the independence of the random effects. With eigenvector spatial filtering, the Moran’s *I* of the neighborhood-level residual declines from 0.101 to 0.005 and becomes non-significant (p = 0.824).

According to the regression results, self-rated health status is significantly higher for respondents meeting the following conditions: male; employed; higher monthly household income; lower stress level; living in a neighborhood with lower KDI and proportionally more physicians. These findings are similar to those of previous studies [23,40-45]. For the doctor-to-resident ratio variable, however, Matteson et al. reported conversely that counties with more family practitioners per capita have higher infant mortality [1]. However, they also found that more hospital beds per capita predicted lower risk of infant death. These results are somewhat contradictory because it is generally considered that the numbers of physicians and hospital beds tend to have a strong positive relationship [46,47]. There does not appear to be a clear and consistent effect of the doctor-to-resident ratio on individual health outcomes; further studies are therefore needed. The present study finds no significant relationship between health status and LGFI, whereas some previous domestic studies reported positive relationship between LGFI and health outcomes [48,49].

This study has several limitations that should be considered in future research. First, even after introducing neighborhood-level variables into the model, variance at neighborhood-level still remains. This may be because some of the key determinants of self-rated health status are omitted. In future research, other neighborhood socioeconomic and environmental factors should be considered to explain the remaining variance. For environmental factors such as air pollution, it is possible to use the interpolated map data in multilevel modeling by integrating it with survey datasets via geographic information science (GIS) [50]. Second, given that the respondents in this study are elderly (aged 60 and over), the employment status variable used in this study can be problematic, because people in their 70s or older are more likely to retire than people in their 60s. In other words, it is possible that the regression result could be confounded by an ‘age’ factor. Third, although census tract data are the only viable option in this study, it could be unclear whether census tracts accurately represent the geographical areas where health-related activities actually occur [21,51]. If they do not, then the estimation of neighborhood effects via these administrative units would be unclear. Due to human mobility, individual health outcomes may be influenced by more complex geographical and temporal contexts beyond their residential environment [52]. However, it is actually difficult to delineate these complex contexts because there is a lack of spatial and temporal information in many cases [51]. Kwan defined this as the uncertain geographic context problem (UGCoP) [51]. To obtain more realistic results, future studies should attempt to identify the actual contexts influencing individual health and mitigate UGCoP. Lastly, some recent studies notice that an approach of removing spatial dependency should practice caution in some cases where neighborhood characteristics change abruptly across a study area. Some researchers have begun to examine this issue; so it must be left to future research.

## Conclusion

This study explores the effects of individual- and neighborhood-level factors on self-rated health status of people over the age of 60 via an approach that combines a multilevel model and an eigenvector spatial filtering technique. The findings show that sex, employment status, monthly household income, and perceived levels of stress are significantly associated with self-rated health status. In addition, residents living in neighborhoods with low deprivation and a high doctor-to-resident ratio tend to report higher health status. There are no changes in the signs of parameters or the significance level between the two models used in this case study. Nevertheless, the proposed spatially filtered multilevel model provides unbiased and robust estimations and has greater explanatory power than conventional multilevel models. The spatially filtered approach is a useful tool for understanding the spatial dynamics of self-rated health status within a multilevel framework. In future research, it would be useful to apply the spatially filtered multilevel model to other datasets in order to clarify the differences between the two models. The inherent modeling complexities of the eigenvector spatial filtering method mean this approach has only recently been put to practical use despite its advantage of visualizing underlying spatial structures. This study hopes that applied models using the eigenvector spatial filtering might be developed in many future studies. Finally, it is hoped that the present findings might inform policy interventions to mitigate health inequality in South Korea.

## Endnote

^a^See the study by Kang et al. [27].

^b^Moran’s *I*, developed by Moran [53], is calculated as follows:

$$I=\frac{n}{\sum_{i}\sum_{j}v_{\mathrm{ij}}}\frac{\sum_{i}\sum_{j}v_{\mathrm{ij}}\left(y_{i}-\bar{y}\right)\left(y_{j}-\bar{y}\right)}{\sum_{i}{\left(y_{i}-\bar{y}\right)}^{2}}$$

where *n* is the number of spatial units; *y*_*i*_ and *y*_*j*_ are attribute values at spatial units *i* and *j*; $\bar{y}$ is the average of *y*; and *v*_*ij*_ is an entity of a spatial weight matrix. If attribute values at *i* and *j* are both higher (or both lower) than the average, Moran’s *I* is a positive value between 0 and 1. When the Moran’s *I* is 1, the attribute values of *i* and *j* are assumed to be perfectly correlated. On the other hand, if the attribute value at *i* is higher than the average, but the value at *j* is lower than the average, the Moran’s *I* is negative. If attribute values of spatial units are perfectly dispersed, Moran’s *I* is -1. A Moran’s *I* of zero indicates that there is no spatial dependency and thus observations are randomly distributed.

## Abbreviations

SAR: Simultaneous autoregressive; GWR: Geographically weighted regression; CHS: Community Health Survey; KDI: Korean Deprivation Index; EQ-5D: EuroQol-5 Dimension; LGFI: Degree of the Local Governments’ Financial Independence; ICC: Intra-class Correlation Coefficient; AIC: Akaike Information Criterion; GIS: Geographic information system; UGCoP: The uncertain geographic context problem.

## Competing interests

The authors declare that they have no competing interests.

## Authors' contributions

YMP contributed to the design of the study, carried out all analyses, drafted the manuscript, and was involved in interpreting research results. YK participated in the design of the study and critically revised the drafted manuscript for important intellectual content. Both authors read and approved the final manuscript.

## References

[B1] Matteson DW Burr JA Marshall JR Infant mortality: a multi-level analysis of individual and community risk factors Soc Sci Med 1998 47 1841 1854 10.1016/S0277-9536(98)00229-9 9877352

[B2] O'Campo P Xue X Wang MC Caughy M Neighborhood risk factors for low birthweight in Baltimore: a multilevel analysis Am J Public Health 1997 87 1113 1118 10.2105/AJPH.87.7.1113 9240099 PMC1380883

[B3] Ahern J Pickett KE Selvin S Abrams B Preterm birth among African American and white women: a multilevel analysis of socioeconomic characteristics and cigarette smoking J Epidemiol Community Health 2003 57 606 611 10.1136/jech.57.8.606 12883067 PMC1732558

[B4] McLafferty S Wang F Luo L Butler J Rural–urban inequalities in late-stage breast cancer: spatial and social dimensions of risk and access Environment and Planning B: Planning and Design 2011 38 726 740 10.1068/b36145 PMC3547633 23335830

[B5] Drukker M Kaplan C Feron F van Os J Children's health-related quality of life, neighbourhood socio-economic deprivation and social capital: a contextual analysis Soc Sci Med 2003 57 825 841 10.1016/S0277-9536(02)00453-7 12850109

[B6] Oren E Koepsell T Area-based socio-economic disadvantage and tuberculosis incidence Int J Tuberc Lung Dis 2012 16 880 885 10.5588/ijtld.11.0700 22583660

[B7] Jerrett M Gale S Kontgis C Spatial modeling in environmental and public health research Int J Environ Res Public Health 2010 7 1302 1329 10.3390/ijerph7041302 20617032 PMC2872363

[B8] Cakmak S Burnett R Spatial regression models for large-cohort studies linking community air pollution and health Journal of Toxicology and Environmental Health, Part A: Current Issues 2003 66 1811 1824 10.1080/15287390306444 12959845

[B9] Chakraborty J Maantay J, McLafferty S Revisiting Tobler’s first law of geography: spatial regression models for assessing environmental justice and health risk disparities Geospatial analysis of environmental health 2011 4 2011 Dordrecht: Springer 337 356

[B10] Corrado L Fingleton B Multilevel modelling with spatial effect 2011 Glasgow: University of Strathclyde press

[B11] Xu H Compare spatial and multilevel regression models for binary outcomes in neighborhood studies Sociological Methodology, forthcoming 10.1177/0081175013490188 PMC4181593 25284905

[B12] Langford IH Leyland AH Rasbash J Goldstein H Multilevel modelling of the geographical distributions of diseases J R Stat Soc: Ser C: Appl Stat 1999 48 253 268 10.1111/1467-9876.00153 12294883

[B13] Fotheringham AS Brunsdon C Charlton M Geographically Weighted Regression: the analysis of spatially varying relationships 2002 England: John Wiley & Sons

[B14] Lorant V Thomas I Deliege D Tonglet R Deprivation and mortality: the implications ofspatial autocorrelation for health resources allocation Soc Sci Med 2001 53 1711 1719 10.1016/S0277-9536(00)00456-1 11762895

[B15] Pickett KE Multilevel analyses of neighbourhood socioeconomic context and health outcomes: a critical review J Epidemiol Community Health 2001 55 111 122 10.1136/jech.55.2.111 11154250 PMC1731829

[B16] Morenoff JD Neighborhood mechanisms and the spatial dynamics of birth weight Am J Sociol 2003 108 976 1017 10.1086/374405 14560732

[B17] Chen DR Wen TH Elucidating the changing socio-spatial dynamics of neighborhood effects on adult obesity risk in Taiwan from 2001 to 2005 Health & Place 2010 16 1248 1258 10.1016/j.healthplace.2010.08.013 20832348

[B18] Chen DR Truong K Using multilevel modeling and geographically weighted regression to identify spatial variations in the relationship between place-level disadvantages and obesity in Taiwan Appl Geogr 2012 32 737 745 10.1016/j.apgeog.2011.07.018

[B19] Wheeler D Tiefelsdorf M Multicollinearity and correlation among local regression coefficients in geographically weighted regression J Geogr Syst 2005 7 161 187 10.1007/s10109-005-0155-6

[B20] Griffith DA Spatial autocorrelation and spatial filtering 2003 New York: Springer

[B21] Patuelli R Schanne N Griffith DA Nijkamp P Persistence of regional unemployment: application of a spatial filtering approach to local labor markets in Germany J Reg Sci 2012 52 300 323 10.1111/j.1467-9787.2012.00759.x

[B22] Griffith DA A comparison of four analytical disease mapping techniques as applied to West Nile Virus in the coterminous Uited States Int J Health Geogr 2005 4 18 10.1186/1476-072X-4-18 16076391 PMC1215506

[B23] Yoon TH Regional health inequalities in Korea: the status and policy tasks J Crit Soc Policy 2010 30 49 77

[B24] EuroQol - Home http://www.euroqol.org/

[B25] Group EQ EuroQol - a new facility for the measurement of health related quality of life Health Policy 1990 16 199 208 10109801 10.1016/0168-8510(90)90421-9

[B26] Kind P Spilker B The EuroQol instrument: an index of Health-related Quality of Life Quality of Life and Pharmacoeconomics in Clinical Trials 1996 2 Philadelphia: Lippincott-Raven 191 201

[B27] Kang E Shin H Park H Jo M Kim N A valuation of health status using EQ-5D Korean J Health Econ Policy 2006 12 19 43

[B28] Schelling TC Models of segregation Am Econ Rev 1969 59 488 493

[B29] Schelling TC Dynamic models of segregation J Math Sociol 1971 1 143 186 10.1080/0022250X.1971.9989794

[B30] Elio & Company Health Ranking 2011 Seoul: Elio & Company

[B31] Kreft IGG de Leeuw J Introducing multilevel modeling 1998 London: Sage

[B32] Luke DA Multilevel modeling 2004 Thousand Oaks: Sage

[B33] Griffith DA A linear regression solution to the spatial autocorrelation problem J Geogr Syst 2000 2 141 156 10.1007/PL00011451

[B34] Haining R Spatial data analysis: theory and practice 2003 Cambridge: Cambridge University press

[B35] Tiefelsdorf M Griffith DA Semiparametric filtering of spatial autocorrelation: the eigenvector approach Environment and Planning A 2007 39 1193 1221 10.1068/a37378

[B36] Chun Y Modeling network autocorrelation within migration flows by eigenvector spatial filtering J Geogr Syst 2008 10 317 344 10.1007/s10109-008-0068-2

[B37] Bates D Maechler M Bolker B lme4: Linear mixed-effects models using S4 Classes 2013 http://cran.r-project.org/web/packages/lme4/index.html. [R package version 0.999999-2

[B38] Bivand R spdep: Spatial dependence: weighting schemes, statistics and models 2013 http://cran.r-project.org/web/packages/spdep/index.html. [R package version 0.5-57

[B39] Akaike H Factor analysis and AIC Psychometrika 1987 52 317 332 10.1007/BF02294359

[B40] Hanyang University Industry Academic Cooperation Foundation Management center for health promotion: Health promotion strategies and programmes development for health inequalities alleviation 2009 Seoul: Ministry of Health and Welfare

[B41] Carstairs V Morris R Deprivation: explaining differences in mortality between Scotland and England and Wales Biritish Med J 1989 299 886 889 10.1136/bmj.299.6704.886 PMC1837760 2510878

[B42] Sloggett A Joshi H Higher mortality in deprived areas: community or personal disadvangate? BMJ 1994 309 1470 1474 10.1136/bmj.309.6967.1470 7804047 PMC2541648

[B43] Davey Smith G Hart CL Watt G Hole DJ Hawthorne VM Individual social class, area-based deprivation, cardiovascular disease risk factors, and mortality: the Renfrew and Paisley study J Epidemiol Community Health 1998 52 399 405 10.1136/jech.52.6.399 9764262 PMC1756721

[B44] Byun YC Regional differences in health expectancy in Korea and policy suggestions 2011 The Korea Institute for Health and Social Affairs: Seoul

[B45] Han MA Ryu SY Park J Kang MG Park JK Kim KS Health-related Quality of Life assessment by the EuroQol-5D in some rural adults J Prev Med Public Health 2008 41 173 180 10.3961/jpmph.2008.41.3.173 18515994

[B46] Goodman DC Fisher ES Bronner KK Hospital and physician capacity update: a brief report from the Dartmouth Atlas of health care 2009 Dartmouth Institute for Health Policy and Clinical Practice: Hanover 36375004

[B47] Leu RE Rutten FFH Brouwer W Matter P Rütschi C The Swiss and Dutch health insurance systems: universal coverage and regulated competitive insurance markets http://www.commonwealthfund.org/Publications/Fund-Reports/2009/Jan/The-Swiss-and-Dutch-Health-Insurance-Systems--Universal-Coverage-and-Regulated-Competitive-Insurance.aspx

[B48] Jo DG A spatial analysis of sociodemographic correlates of Health related Quality of Life Korean J Popul Stud 2009 32 1 20

[B49] Han JY Na BJ Lee MS Hong JY Lim NG The relationship between local fiscal indices and standardized mortality rate Proceedings of the KAIS 2010 Fall conference: 12-13 November 2010; Jeju 2010 Cheonan: The Korea Academia-Industrial cooperation Society 1072 1076

[B50] Root E Emch M Maantay J, McLafferty S Regional environmental patterns of diarrheal disease in Bangladesh: a spatial analytical and multilevel approach Geospatial analysis of environmental health. Volume 4 2011 2011 Dordrecht: Springer 191 204

[B51] Kwan M-P The uncertain geographic context problem Annals of the Association of American Geographers 2012 102 958 968 10.1080/00045608.2012.687349

[B52] Gatrell AC Mobilities and health 2011 Aldershot: Ashgate

[B53] Moran PAP Notes on continuous stochastic phenomena Biometrika 1950 37 17 23 15420245

